# Robust entanglement between a movable mirror and atomic ensemble and entanglement transfer in coupled optomechanical system

**DOI:** 10.1038/srep33404

**Published:** 2016-09-14

**Authors:** Cheng-Hua Bai, Dong-Yang Wang, Hong-Fu Wang, Ai-Dong Zhu, Shou Zhang

**Affiliations:** 1 Department of Physics, College of Science, Yanbian University, Yanji, Jilin 133002, China

## Abstract

We propose a scheme for the creation of robust entanglement between a movable mirror and atomic ensemble at the macroscopic level in coupled optomechanical system. We numerically simulate the degree of entanglement of the bipartite macroscopic entanglement and show that it depends on the coupling strength between the cavities and is robust with respect to the certain environment temperature. Inspiringly and surprisingly, according to the reported relation between the mechanical damping rate and the mechanical frequency of the movable mirror, the numerical simulation result shows that such bipartite macroscopic entanglement persists for environment temperature up to 170 K, which breaks the liquid nitrogen cooling and liquid helium cooling and largely lowers down the experiment cost. We also investigate the entanglement transfer based on this coupled system. The scheme can be used for the realization of quantum memories for continuous variable quantum information processing and quantum-limited displacement measurements.

Entanglement, the characteristic trait of quantum mechanics^1^, has appealed widespread attention and interest in different branches of physics and has also become the essential resource for many quantum information processes^2^,^3^. Up to now, entanglement has been successfully prepared and manipulated in various microscopic systems theoretically and experimentally, such as atoms^4^,^5^,^6^,^7^,^8^, photons^9^,^10^,^11^, ions^12^,^13^,^14^, and so on. However, it is not yet completely clear that to what degree quantum mechanics is suitable for mesoscopic and macroscopic systems^15^. Although quantum mechanics has proven to be highly successful in explaining physics at microscopic and subatomic scales, its validity at macroscopic or even mesoscopic scales is still debated. Some of the astonishing features appear when we try to apply quantum-mechanical principles to macroscopic systems. Quantum phenomenon such as entanglement of macroscopic systems is one example. In the principles of quantum mechanics, the preparation of entanglement in mesoscopic and macroscopic systems should not be violated^15^,^16^,^17^, but entanglement generally does not appear in the macroscopic world resulting from environment-induced decoherence. Therefore, it is of crucial significance to investigate the possibilities to obtain entangled states of macroscopic systems and to study the robustness of entanglement against temperature. Many scientists tried their best to observe this novel phenomenon of quantum entanglement at the macroscopic level over the last decades. Fortunately, due to the deepening of theoretical investigation and the advancement of experimental techniques, obtaining and observing entanglement in mesocopic and even macroscopic systems have become possible.

With the fast-developing fields of microfabrication and nanotechnology, cavity optomechanical system is being one of the most appealing and promising candidates as an ideal system for the study of fundamental quantum physics, such as macroscopic quantum phenomena, decoherence, and quantum-classical boundary^18^. Its standard model consists of a Fabry-Pérot cavity with one fixed partially transmitting mirror and one movable perfectly reflecting mirror. When the Fabry-Pérot cavity is coherently driven by an external laser field, the movable mirror will be shifted from its equilibrium position and free to move along the cavity axis due to the radiation pressure force and its center-of-mass motion can be modelled as a mechanical harmonic oscillator. Since the mechanical harmonic oscillator is a typical represent of classical systems^15^, cavity optomechanical system provides a unique platform for exploring the novel quantum phenomena at the macroscopic level such as quantum entanglement. In recent years, a number of schemes covering this topic have been proposed based on the cavity optomechanical system^15^,^19^,^20^,^21^,^22^,^23^,^24^,^25^,^26^,^27^,^28^. In 2007, Vitali *et al*. theoretically investigated stationary entanglement between an optical cavity field mode and a macroscopic vibrating mirror in a standard optomechanical setup and showed that such optomechanical entanglement was robust against the environment temperature above 20 K^15^. Latter, their group also realized the tripartite and bipartite continuous variable entanglement by placing an ensemble of two-level atoms inside the Fabry-Pérot cavity^19^. Then they investigated in detail the entanglement properties between the experimentally detectable output field of an optical cavity and a vibrating cavity end-mirror^20^. In 2012, Joshi *et al*. theoretically investigated the possibility of generating nonlocal quantum entanglement between optical and mechanical modes of two spatially separated cavities which are coupled by an optical fiber^21^. Also in 2012, Akram *et al*. considered the entanglement between the different optical and mechanical modes in an array of three optomechanical cavities which are coupled either reversibly or irreversibly to each other^22^. In 2013, Ge *et al*. investigated the entanglement transfer from two-mode fields to two movable mirrors via placing the gain medium of cascading three-level atoms inside a doubly resonant cavity^23^. In 2014, Liao *et al*. proposed a scheme to generate quantum entanglement between two macroscopic mechanical resonators in a two-cavity optomechanical system^24^. In 2015, Huan *et al*. theoretically investigated entanglement transfer from the intracavity photon-phonon entanglements to an intercavity photon-photon entanglement in a double-cavity system^25^. Wu *et al*. investigated the entanglement properties in a hybrid system consisting of an optical cavity-array coupled to a mechanical resonator in 2015^26^. Also in 2015, Li *et al*. investigated the entanglement between two movable mirrors in an optomechanical cavity^27^. We note that entanglement between two macroscopic mechanical resonators in a two-cavity coupled optomechanical system has been investigated^24^ and entanglement between two movable mirrors in an optomechanical cavity has also been investigated^27^, while entanglement between an atomic ensemble and a movable mirror in an optomechanical cavity has been investigated earlier^19^. However, to our knowledge, the entanglement between the atomic ensemble and the movable mirror in a two-cavity coupled optomechanical system has not been investigated. So, a natural question is that whether there exists quantum entanglement between the atomic ensemble and the movable mirror in a two-cavity coupled system except for between the macroscopic mechanical resonators. In addition, some potential technologies of the coupled cavities in experiment have been demonstrated^29^,^30^. The coupled system is thought to be suitable for building a large-scale architecture for quantum information processing. Recently, much attention has also been focused on the coupled optomechanical cavities system^31^,^32^,^33^. These observations remind us of the necessity to explore the entanglement properties of the atomic ensemble-mirror in a two-cavity coupled optomechanical system. Here we propose a scheme for the creation of robust entanglement between the atomic ensemble and a movable mirror at the macroscopic level in coupled optomechanical system. In the scheme, with the increase of the coupling strength of the coupled optomechanical system, not only the entanglement is increasingly stronger but also the region of the effective detuning that entanglement exists is more and more broader, which are extremely significant due to the fact that the stronger entanglement and the more broader effective detuning region are obtained, the more easily it is realized and observed in experiment. Meanwhile, in experimentally accessible parameter regimes, the numerical simulation result indicates that critical temperature of the bipartite macroscopic entanglement can up to at least 32 K, higher than that in refs 15, 19, 27 and 28. Very fortunately, we find that the relation between the mechanical damping rate and the mechanical frequency of the movable mirror *γ*_*m*_ = 10^−6^ *ω*_*m*_ has been reported^34^. In this case, when the coupling strength between the coupled optomechanical cavities is set to be *J* = *ω*_*m*_, the critical temperature can be increased to 170 K, which breaks the liquid nitrogen cooling and liquid helium cooling and will largely lower down the experiment cost. Moreover, we also investigate the entanglement transfer based on this coupled system. Our scheme can be used for the realization of quantum memories for continuous variable quantum information processing and quantum-limited displacement measurements.

The remainder of this paper is organized as follows. In Sec. II we establish the theoretical model of the coupled optomechanical system and present the equations of motion of the system. In Sec. III we quantify the entanglement properties of the system by introducing the logarithmic negativity. In an experimentally accessible parameter regime, we simulate the entanglement properties of the coupled optomechanical system numerically in Sec. IV. Finally we make a conclusion to summarize our results in Sec. V.

## Results

### The coupled optomechanical system model and equations of motion

**Figure 1:**
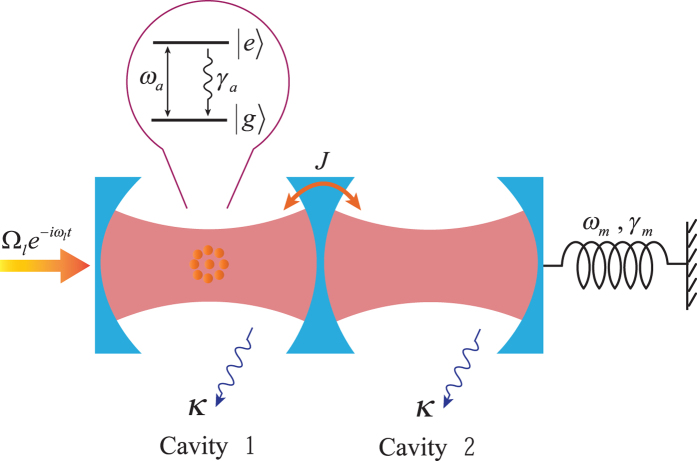
Schematic illustration of the coupled optomechanical system including cavity 1 coupled to cavity 2 with coupling strength *J*. An ensemble of two-level atoms is placed into the cavity 1 which is coherently driven by an external monochromatic laser field with strength Ω_*l*_ and frequency *ω*_*l*_. The vibrational motion of the oscillating mirror for cavity 2 can be modelled as a mechanical harmonic oscillator of frequency *ω*_*m*_ and decay rate *γ*_*m*_ and is shifted from the equilibrium position due to the radiation pressure force.

As schematically shown in Fig. 1, the system studied here is composed of two coupled single-mode cavities and an ensemble of two-level atoms. Cavity 1 contains the atomic ensemble and is coherently driven by an external monochromatic laser field with strength Ω_*l*_ and frequency *ω*_*l*_ and cavity 2 with a fixed mirror and a second oscillating mirror couples to the cavity 1 with the coupling strength *J*. The optical field of cavity 2 is coupled to the mechanical motion of the movable mirror via radiation pressure force and mirror vibrational motion can be modelled as a mechanical harmonic oscillator of frequency *ω*_*m*_ and decay rate *γ*_*m*_. Experimentally, such a double-cavity optomechanical model can be carried out in the systems based on Fabry-Pérot cavities or whispering-gallery cavities^35^,^36^,^37^. The Hamiltonian for describing the coupled optomechanical system is written as^38^


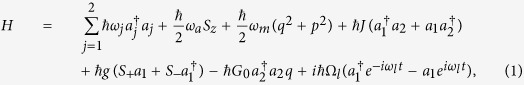


where *a*_*j*_ is the bosonic operator eliminating a photon in the *j*-th cavity with resonance frequency *ω*_*j*_. The atomic ensemble is composed of *N* two-level atoms with intrinsic frequency *ω*_*a*_ each described by the spin-1/2 Pauli matrices *σ*_+_, *σ*_−_, and *σ*_*z*_. Collective spin operators are defined as 
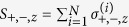
 and satisfy the commutation relations [*S*_+_, *S*_−_] = *S*_*z*_ and [*S*_*z*_, *S*_±_] = ±2*S*_±_. *q* and *p* are the dimensionless position and momentum operators of the oscillating mirror, respectively, and satisfy [*q*, *p*] = *i*. *g* is the atom-cavity coupling constant and given by 
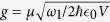
, where *μ* is the dipole moment of the atomic transition, *ϵ*_0_ is the free space permittivity, and *V* is the volume of cavity 1 mode. 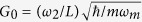 is the radiation pressure coupling strength, with *L* the cavity length in the absence of the intracavity field and *m* the effective mass of the mechanical mode^39^. The strong drive of amplitude 
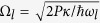
, with *P* and *κ* the drive laser input power and the cavity decay rate, respectively, resulting in a large steady-state optical field in the cavity, which increases the occupation numbers in each mode and the radiation pressure coupling. The induced steady-state intracavity in turn shifts the equilibrium position of the mechanical oscillator via the radiation pressure force. In Eq. (1), the first three terms denote the free energy of the coupled optomechanical system, the fourth term represents the coupling between the cavity 1 and cavity 2, the fifth term describes the coupling of atomic ensemble with cavity mode, the sixth term represents the coupling of optical mode with mechanical mode, and the last term describes the coupling of laser drive with the cavity, respectively.

We consider a compact scenario of such a equation, which is accessible in the low atomic excitation limit, i.e., the average number of atoms in the excited state |*e*〉 is much smaller than the number of total atoms^40^. In this limit, the collective spin operators *S*_±_, *S*_*z*_ of the atomic polarization can be described in terms of the bosonic annihilation and creation operators *c* and *c*^†^ via the Holstein-Primakoff transformation^41^,^42^


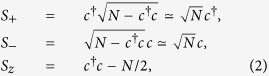


where the usual bosonic commutation relation [*c*, *c*^†^] = 1 is still satisfied.

Transforming the above Hamiltonian into the rotating frame at the frequency *ω*_*l*_ of the driving laser field, we rewrite the system Hamiltonian as


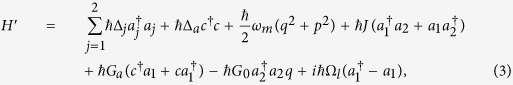


where Δ_*j*_ = *ω*_*j*_ − *ω*_*l*_ and Δ_*a*_ = *ω*_*a*_ − *ω*_*l*_ are, respectively, the cavity mode and atomic detuning with respect to the driving laser, 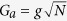.

A proper analysis of the dynamics of the coupled optomechanical system can be accomplished by a set of nonlinear Langevin equations in which the corresponding dissipation and fluctuation terms are added to the Heisenberg equations of motion derived form the Eq. (3)


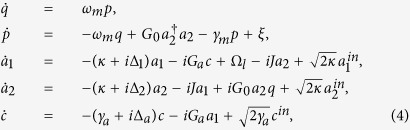


where *γ*_*a*_ is the decay rate of the atomic excited state level and the nonvanishing correlation functions of noises affecting atoms and cavity fields obey the relations 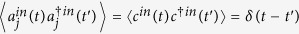
43,44. Here we have assumed that the cavity 1 and cavity 2 have the same decay rate *κ*. Furthermore, the mechanical mode is also affected by the stochastic Hermitian Brownian noise *ξ* that satisfies the non-Markovian correlation function with a colored spectrum in general^44^


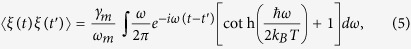


where *k*_*B*_ is the Boltzmann constant and *T* is the temperature of the mechanical oscillator. However, quantum effects are revealed only for the mechanical oscillator with a high quality factor, i.e., 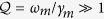. In this limit, this non-Markovian process can be approximated as a Markovian one and the Brownian noise *ξ*(*t*) can be simplified to delta-correlated^45^,^46^


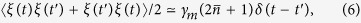


where 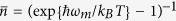 is the mean thermal excitation number. In the following we discuss the entanglement of the coupled optomechanical system in the regime where the system is stable.

### The steady-state entanglement of the coupled optomechanical system

We now begin to linearize the dynamics of the coupled optomechanical system. The nonlinear quantum Langevin equations can be linearized by rewriting each Heisenberg operator as a sum of its steady-state mean value and an additional fluctuation operator with zero-mean value, i.e., *q* = *q*_*s*_ + *δq*, *p* = *p*_*s*_ + *δp*, *a*_*j*_ = *a*_*js*_ + *δa*_*j*_ (*j* = 1, 2), and *c* = *c*_*s*_ + *δc*^47^. Substituting these expressions into Eq. (4) and the latter will be separated into a set of nonlinear algebra equations for the steady-state value and a set of quantum Langevin equations for the fluctuation operators^48^. The steady-state mean values of the coupled optomechanical system can be obtained by setting the time derivatives to zero,


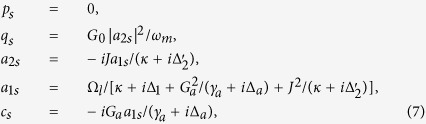


where 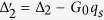 is the effective detuning of the second cavity mode.

We assume that the cavity is intensively driven with a very large input power *P*, so that at the steady state, the intracavity fields have a large amplitude *a*_*js*_, i.e., 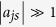. In the strong driving limit, we can safely omit the nonlinear quantities 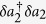 and *δa*_2_*δq* and get the following linearized Langevin equations,


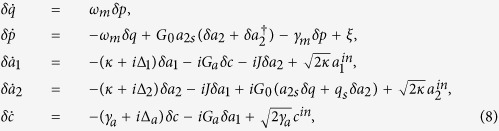


where we have chosen the phase reference of the cavity fields so that *a*_*js*_ can be taken real. Here we will devote to establishing the presence of quantum correlations among the subsystems of the coupled optomechanical system at the steady state, which can be carried out by analyzing the dynamics of the quantum fluctuations of the coupled optomechanical system around the steady state. The resulting evolution equations of motion for the fluctuations in Eq. (8) can be rewritten in a compact form as follows,


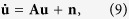


where the vector of eight-component quadrature fluctuations 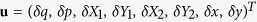, similarly the input-noise vector 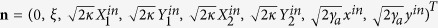, here 
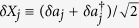
, 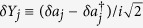, 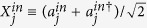, 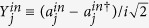 (*j* = 1, 2), 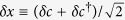, 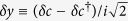, 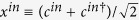, 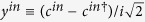 and the drift matrix **A** is given by


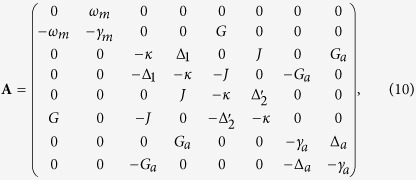


where 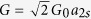 is the effective optomechanical coupling. Now the quantum fluctuations of the field and the oscillating mirror are coupled by the much larger effective optomechanical coupling *G*, so the engineering of significant optomechanical entanglement in coupled system becomes possible^15^.

The linearized effective Hamiltonian which corresponds to the linearized Langevin Eq. (8) ensures that when the system is stable, it always reaches a Gaussian state whose information-related properties, such as entanglement and entropy, can be completely described by the symmetric 8 × 8 covariance matrix **V** whose element 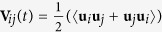 for *i*, *j*= 1, 2, …, 8. The coupled system is stable and reaches its steady state only if the real part of all the eigenvalues of the drift matrix **A** are negative. The stability conditions can be derived by applying the Routh-Hurwitz criterion^49^ and the case of eight dimensions matrix is shown in ref. 23. We will guarantee the stability conditions of the system in the following analysis. When the stability conditions of the coupled system are satisfied, the steady-state correlation matrix can be derived from the following Lyapunov equation^15^,^50^,^51^


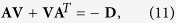


where 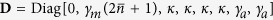 is the diagonal matrix for the corresponding damping and leakage rates stemming from the noise correlations.

From this equation, the covariance matrix **V** can be written as the form of a block matrix


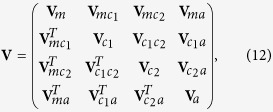


where each block represents 2 × 2 matrix. The blocks on the diagonal indicate the variance within each subsystem (the oscillating mirror, the cavity mode 1, the cavity mode 2, and the atomic ensemble), while the off-diagonal blocks indicate covariance across different subsystems, i.e., the correlations between two components of the whole coupled optomechanical system.

To compute the entanglement among the subsystems of the coupled optomechanical system, we reduce the 8 × 8 covariance matrix **V** to a 4 × 4 submatrix **V**_*S*_. If the indices *i* and *j* for the element **V**_*ij*_ are confined to the set {1, 2, 3, 4}, the submatrix **V**_*S*_ = [**V**_*ij*_] is formed by the first four rows and columns of **V** and corresponds to the covariance between the cavity 1 mode and the oscillating mirror. Similarly, if the indices run over {1, 2, 5, 6}, **V**_*S*_ is the covariance matrix of the cavity mode 2 and the oscillating mirror. If the indices run over {1, 2, 7, 8}, **V**_*S*_ labels the covariance between the atomic ensemble and the oscillating mirror. Summarizing, the submatrix can be written as


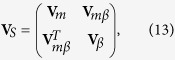


where *m* and *β* index the subsystem {oscillating mirror, cavity 1 (cavity 2, atomic ensemble)} in the coupled optomechanical system.

Next we resort to Simon’s criterion to judge continuous variable entanglement^52^. For a physical state, the covariance matrix **V** must obey the Robertson-Schrödinger uncertainty principle


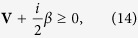


where 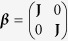 with 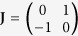. Here we define the vector **F** = (*Q*_1_, *P*_1_, *Q*_2_, *P*_2_)^*T*^ for a two-mode system. If a state is separable, partial transpose matrix 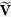 (obtained from **V** just by taking *P*_*j*_ in −*P*_*j*_) still comply with the Eq. (14). This inequality equation requires that all the symplectic eigenvalues of the transposed matrix to be larger than 1/2. So if the smallest eigenvalues is less than 1/2, the transposed modes are inseparable, i.e., there exists entanglement between the modes. We introduce the logarithmic negativity to quantify the entanglement which can be computed by means of a process known as symplectic diagonalization of submatrix **V**_*S*_, where the entanglement properties are characterized in the symplectic eigenvalues of the diagonalized matrix. If the diagonalized matrix is written as Diag[*ν*_−_, *ν*_−_, *ν*_+_, *ν*_+_], then the eigenvalues along the diagonal is ref. 53


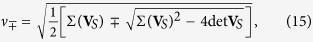


where 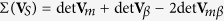. We regard *ν*_−_ as the minimum sysplectic eigenvalue of the covariance matrix and the logarithmic negativity *E*_*N*_ can be defined as *E*_*N*_ = max[0, −ln2*ν*_−_]^54^. Therefore, the symplectic eigenvalue *ν*_−_ completely quantifies the quantum entanglement among the subsystems and they are entangled if and only if 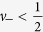, which is consistent with the Simon’s criterion. In the next section, we utilize the logarithmic negativity *E*_*N*_ to show the entanglement properties of the coupled optomechanical system numerically.

## Discussion

In the following, we investigate the stationary optomechanical entanglement among the subsystems numerically. In fact, Eq. (11) is a linear equation for **V** and can be straightforwardly solved, but the general exact expression is too tedious. However, it is easy to simulate numerically. In our numerical calculations, we adopt the set of experimental parameters for the coupled optomechanical system given in Table 1, which can be carried out in current experiments^55^,^56^,^57^, consequently, our scheme is experimentally feasible. In order to produce continuous variable entanglement in coupled optomechanical system, we must construct the effective Hamiltonian of the nondegenerate parametric-down conversion type for the system through setting 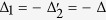
58.

**Figure 2:**
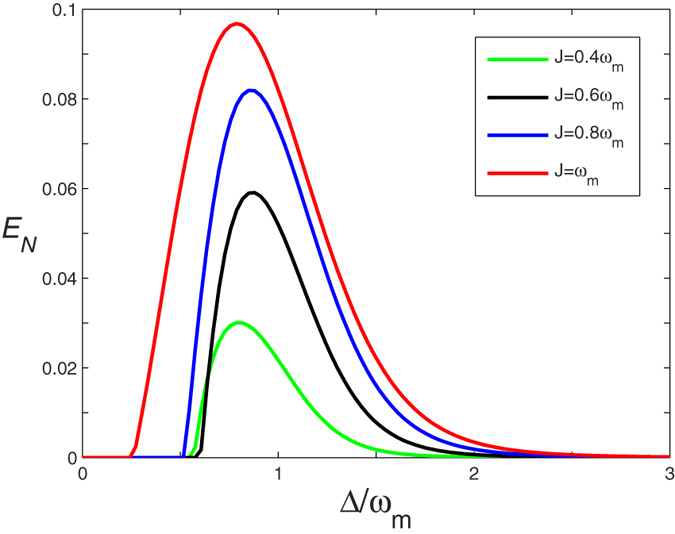
The logarithmic negativity *E*_*N*_ as a function of the normalized detuning Δ/*ω*_*m*_ with four different optical coupling strength *J* at a fixed temperature *T* = 400 mK and the other parameters are given in Table 1.

Firstly, we investigate the entanglement of two indirectly coupled macroscopic objects. In the experimentally accessible parameter regimes, our scheme realizes the robust entanglement between the movable mirror and atomic ensemble (*N* ~ 10^7 ^19,59) in the coupled optomechanical system, which is incredible in the macroscopic world. Figure 2 shows the logarithmic negativity *E*_*N*_ between the movable mirror and atomic ensemble versus the normalized detuning Δ/*ω*_*m*_ for different coupling strengths. It can be clearly seen from Fig. 2 that not only the entanglement is increasingly stronger but also the region of the effective detuning that entanglement exists is more and more broader with the increase of the coupling strength, which are extremely significant due to the fact that the stronger entanglement and the more broader effective detuning region are obtained, the more easily it is realized and observed in experiment. Furthermore, the mirror-atomic ensemble entanglement is present only within a finite interval of values of Δ around 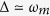, which is in accordance with ref. 15.

**Figure 3:**
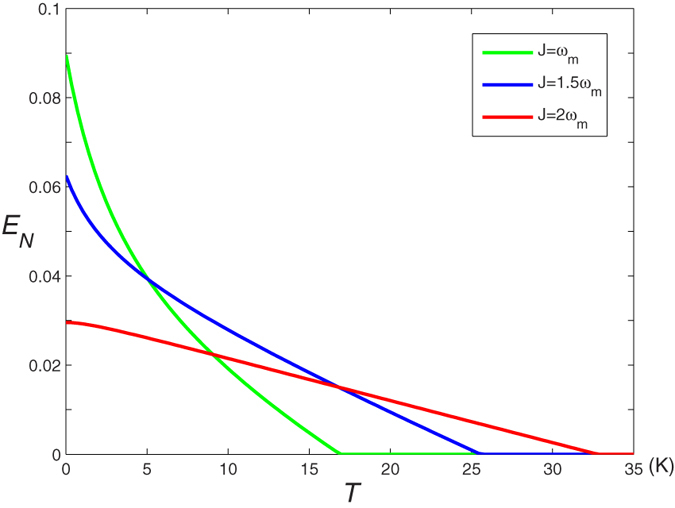
The logarithmic negativity *E*_*N*_ versus the environment temperature *T* with three different optical coupling strength *J*. Here Δ = *ω*_*m*_ and the other parameters are given in Table 1.

**Figure 4:**
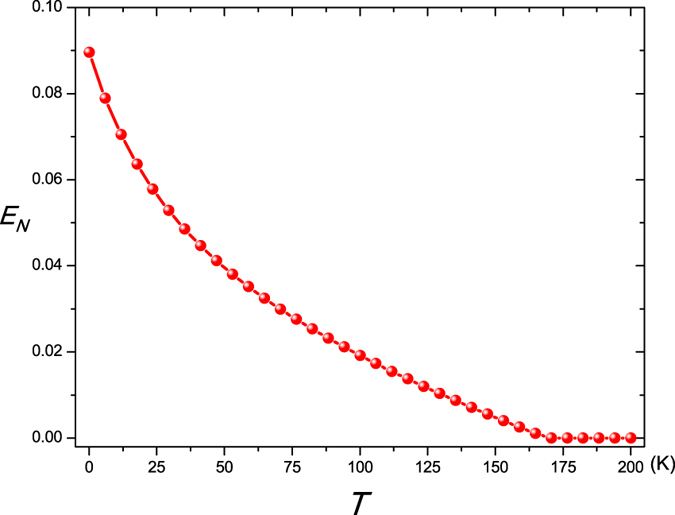
The logarithmic negativity *E*_*N*_ versus the environment temperature *T* when the coupling strength is set to be *J* = *ω*_*m*_. Here Δ = *ω*_*m*_, *γ*_*m*_ = 10^−6^ *ω*_*m*_, and the other parameters are given in Table 1.

The robustness of such a mirror-atomic ensemble entanglement with respect to the environment temperature *T* of oscillating mirror is shown in Fig. 3. As clearly presented in Fig. 3, due to the environment-induced decoherence, the intensity of the mirror-atomic ensemble entanglement decreases and eventually vanishes with the rise of environmental temperature. With the increase of coupling strength *J*, the critical value of temperature *T*_*c*_ (*T*_*c*_ is defined as *T* ≥ *T*_*c*_, *E*_*N*_ = 0) increases. When the coupling strength is set *J* = 2 *ω*_*m*_, the critical value of temperature *T*_*c*_ of the mirror-atomic ensemble entanglement persists for 32 K, which is several orders of magnitude larger than the ground state temperature of the mechanical oscillator and is higher than that in refs 15, 19, 27 and 28. Meanwhile, if the coupling strength is further strengthen^60^, the critical temperature will also be further risen. For example, if the coupling strength is set to be *J* = 2.5 *ω*_*m*_, the critical temperature will increase to 36 K. Very fortunately, we find that the relation between the mechanical damping rate and the mechanical frequency of the movable mirror *γ*_*m*_ = 10^−6^ *ω*_*m*_ has been reported^34^. In this case, when the coupling strength between the coupled optomechanical cavities is set to be *J* = *ω*_*m*_, as shown in Fig. 4, the critical temperature can be increased to 170 K, which breaks the liquid nitrogen cooling and liquid helium cooling and will largely lower down the experiment cost. Therefore, it is easier and more feasible to realize and observe the macroscopic level mirror-atomic ensemble entanglement from the experimental point view.

**Figure 5:**
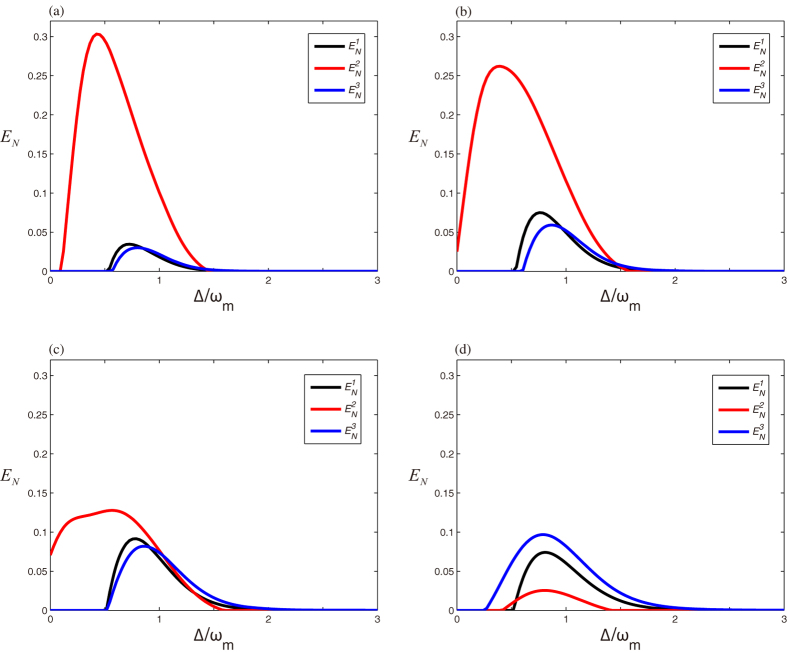
The logarithmic negativity 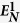 (black curves), 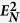 (red curves), and 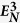 (blue curves) as a function of the normalized detuning Δ/*ω*_*m*_ at a fixed temperature *T* = 400 mK and the other parameters are given in Table 1. (**a**) *J* = 0.4 *ω*_*m*_, (**b**) *J* = 0.6 *ω*_*m*_, (**c**) *J* = 0.8 *ω*_*m*_, (**d**) *J* = *ω*_*m*_.

Secondly, we investigate the stationary entanglement of the three possible bipartite subsystems in terms of the logarithmic negativity *E*_*N*_. We denote the logarithmic negativities for the cavity 1-mirror, cavity 2-mirror, and atomic ensemble-mirror as 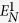, 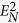, and 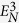, respectively. The bipartite entanglements between the cavity 1-cavity 2, cavity 1-atomic ensemble, and cavity 2-atomic ensemble are so weak that there is no need to consider them. The results on the behavior of the bipartite entanglement are shown in Fig. 5 in which we plot three bipartite logarithmic negativities 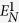
(black curves), 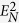
(red curves), and 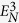
(blue curves) versus the normalized dutuning Δ/*ω*_*m*_ at a fixed temperature of *T* = 400 mK for four different coupling strengths. It is evident that there is a sort of entanglement transfer among the three bipartite subsystems, i.e., the bipartite entanglements 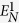 and 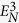 increase while the bipartite entanglement 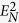 decreases with the increase of coupling strength *J*. In other words, the enhancement of the entanglements between cavity 1 and mirror and atomic ensemble and mirror at the expense of the entanglement between cavity 2 and mirror. It is remarkable that, therefore, the indirectly coupled entanglement (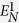 and 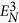) transfers with the directly coupled entanglement 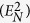 each other with the increase of coupling strength *J*. It is worth noting that in the above discussion the effect of the entanglement transfer among the three bipartite subsystems is predominant when the atoms are resonant with the Stokes sideband (Δ_*a*_ = −*ω*_*m*_). Moreover, we notice that atomic ensemble-mirror entanglement is not present at Δ_*a*_ = *ω*_*m*_, which is due to the fact that the entanglement is mostly carried by the cavity 1-mirror and cavity 2-mirror.

In above discussion, we assume that the average number of atoms in the excited state is much smaller than the number of total atoms. Here we discuss the limits of validity of the model. The bosonic description of the atomic polarization is valid only when the single-atom excitation probability, 
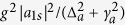
, is much smaller than 1^19^. Furthermore, the linearization of the quantum Langevin equations is valid when the intracavity fields have a large amplitude at the steady state, i.e., 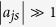. Therefore, the above two conditions are simultaneously satisfied only when 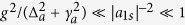. This means requiring a very weak atom-cavity coupling. But if we consider a relatively small cavity mode volume 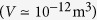, in this case, *g* is not very weak when we consider a standard optical dipole transition. However, the required weak-coupling condition can still be achieved^19^.

We now address the experimental issues. The detection of the generated entanglement at the macroscopic level in optomechanical systems is still an experimental challenge. However, for the detection of the entanglement, we have to measure the quadrature correlations^43^ and quantum correlation detection is relatively easy in optomechanical systems. Recently, several promising programs have been proposed in refs 15, 19, 61 and 62, so we can exploit homodyne measurement experimental techniques to detect quantum correlations so as to detect the indirectly coupled quantum entanglement.

In conclusion, we have proposed a scheme to create robust entanglement between a movable mirror and atomic ensemble at the macroscopic level in coupled optomechanical system. With the increase of the coupling strength of the coupled optomechanical system, the stronger entanglement and the broader effective detuning region can be obtained, so it is easier and more feasible to realize and observe this sort of novel phenomenon in experiment. Utilizing experimentally accessible parameters, the critical temperature of the bipartite macroscopic entanglement in our scheme can approach to 32 K, higher than that in refs 15, 19, 27 and 28. More importantly, according to reported relation between the mechanical damping rate and the mechanical frequency of the movable mirror, the critical temperature can be increased to 170 K, which breaks the liquid nitrogen cooling and liquid helium cooling and will largely lower down the experiment cost. We also investigated the entanglement transfer based on this coupled system. Such a scheme can be used for the realization of quantum memories for continuous variable quantum information processing and quantum-limited displacement measurements.

## Methods

### Derivation of the Langevin equation

To derive the Langevin equation in which the corresponding dissipation and fluctuation terms are added to the Heisenberg equation, we consider a system interacting with a heat bath. The Hamiltonian is given by


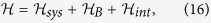


where 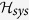 is the free Hamiltonian for the system but we do not specify the concrete form of it, 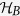 is the free Hamiltonian for the heat bath, and 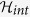 is the interaction Hamiltonian between them. 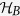 and 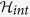 are given respectively


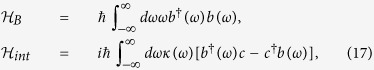


where *b*(*ω*) is boson annihilation operator for the heat bath which satisfies the commutation relation 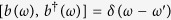, *c* is one of several possible system operators, and *κ*(*ω*) is coupling constant.

From Eqs (16 and 17) we derive the Heisenberg equations of motion for *b*(*ω*) and an arbitrary system operator *a*


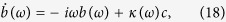



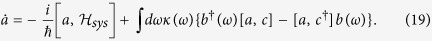


Eq. (18) can be formally integrated as


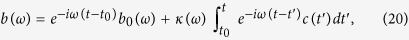


where *b*_0_(*ω*) is the value of *b*(*ω*) at *t* = *t*_0_ and has the same commutation relation as *b*(*ω*). We substitute in Eq. (19) to obtain


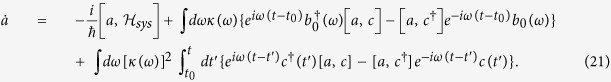


We introduce the first Markov approximation which means the coupling constant is independent of frequency 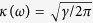. In addition, we define an input field by


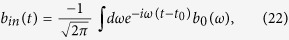


which satisfies the commutation relation 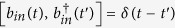. Via using the properties 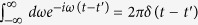 and 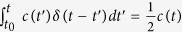, Eq. (21) can derive following Langevin equation


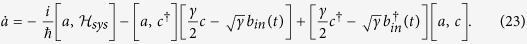


If we specify *c* → *a* and *b*_*in*_(*t*) → *a*_*in*_(*t*), Eq. (23) reduces following form


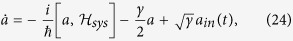


which is a Langevin equation for the damped amplitude *a*(*t*) in which the corresponding dissipation and fluctuation terms are added to the Heisenberg equation.

## Additional Information

**How to cite this article**: Bai, C.-H. *et al*. Robust entanglement between a movable mirror and atomic ensemble and entanglement transfer in coupled optomechanical system. *Sci. Rep.* **6**, 33404; doi: 10.1038/srep33404 (2016).

## Figures and Tables

**Table 1:** The experimental parameters for the coupled optomechanical system used in our numerical simulation, extracted from the experiments in refs 55,56,57.

Systematic parameter	Symbol	Value
Cavity length	*L*	1 mm
Cavity decay rate	*κ*	*π* × 10^7^ Hz
Driven-laser wavelength	*λ*	810 nm
Input laser power	*P*	35 mW
Mechanical frequency	*ω*_*m*_	2*π* × 10^7^ Hz
Mechanical mass	*m*	5 ng
Mechanical damping rate	*γ*_*m*_	200*π* Hz
Atomic decay rate	*γ*_*a*_	*π* × 10^7^ Hz
Atomic ensemble coupling strength	*G*_*a*_	1.2*π* × 10^7^ Hz
